# Mendelian Randomization Identified SLC2A9 as a Novel cis‐eQTL‐Mediated Susceptibility Gene in Suppressing Renal Cancer and Its Related Metabolic Mechanisms

**DOI:** 10.1155/mi/5817314

**Published:** 2026-03-16

**Authors:** Yi Wang, Yu Song, Hao Ji, Bingye Zhu, Huyang Xie

**Affiliations:** ^1^ Department of Urology, Affiliated Hospital of Nantong University, Nantong, 226001, Jiangsu Province, China, ahnmc.com; ^2^ Department of Urology, The Second People’s Hospital of Changzhou, The Third Affiliated Hospital of Nanjing Medical University, Changzhou, 213100, China; ^3^ Department of Urology, Tumor Hospital Affiliated to Nantong University, Nantong University, Nantong, 226300, China, ntu.edu.cn; ^4^ Department of Urology, Affiliated Nantong Hospital of Shanghai University (The Sixth People’s Hospital of Nantong), Nantong, 226001, Jiangsu Province, China

**Keywords:** cis-eQTL, mendelian randomization, metabolites, renal cancer, SLC2A9

## Abstract

**Objective:**

Mendelian randomization (MR) analysis has currently been widely used in combination with expression quantitative trait loci (eQTLs) or cis‐eQTLs for identifying novel drug targets in various diseases. Hence in this article, we first integrated MR analysis with eQTLs and cis‐eQTLs to reveal the causal effects among SLC2A9 and renal cancer (RC), along with its related metabolic mechanisms.

**Methods:**

Related genome‐wide association study (GWAS) data were obtained from online datasets. Two‐sample MR and summary‐data‐based MR study (SMR) analyses were applied to assess the causal effects among SLC2A9 eQTL or cis‐eQTLs and RC, respectively. Sensitivity analysis confirmed the robustness and heterogeneity of the results. Besides, 1400 metabolites were enrolled as mediators to further reveal the related metabolic mechanisms of SLC2A9 eQTL involved in RC.

**Results:**

Our results found that SLC2A9 eQTL was markedly associated with low risks of RC in the IEU OpenGWAS eQTLs dataset (discovering) (*p* = 0.030). The eQTLGEN and GTEx Whole Blood datasets validated the causal effects among SLC2A9 cis‐eQTL and RC, reducing RC risks (both *p*  < 0.05). The TCGA‐KIRC and the CPTAC‐KIRC datasets validated the mRNA and protein expression levels of SLC2A9 in RC, confirming its antitumor roles in RC (both *p*  < 0.05). A total of five metabolites were finally identified to reveal the related metabolic mechanisms of SLC2A9 eQTL involved in RC (all *p*  < 0.05).

**Conclusions:**

This study indicated that SLC2A9 eQTL or cis‐eQTL was markedly associated with low risks of RC and played an antitumor role in RC, along with preliminary identification of five metabolic pathways for its potential mechanisms requiring further investigation.

## 1. Introduction

Renal cancer (RC) was one of the most common kidney tumors in adults worldwide, with ~81,610 estimated new cases and 14,390 estimated new deaths in the United States in 2024 [1]. It could be subdivided into more than 40 subtypes, including clear cell renal cell carcinoma (ccRCC), papillary RCC (pRCC), chromophobe RCC (chRCC), collecting duct RCC (CDRCC), sarcomatoid RCC (srRCC), and so on [2]. Smoking, obesity, hypertension, chronic kidney disease, and so on, were epidemiologically recognized as consistent risk factors for RC [3]. Currently, the mainstay of therapy for treating RC included surgery, targeted therapies, immunotherapy, and so on [4]. Although the 5‐year survival rate for individuals with nonmetastatic RC after surgery was over 80% [5], it remained fewer than 20% for patients with metastatic RC [6]. Therefore, there was an urgent need for us to identify novel therapeutic targets for RC and their potential mechanisms in the era of precision medicine.

Metabolic imbalances were commonly regarded as one of the characteristics of cancer, and the metabolic intermediates that abnormally accumulated in tumor cells had been reported to be strongly linked to the development of various tumors [7]. Zhu et al. [8] identified a significant correlation between hemoglobin synthesis and the progress of pancreatic ductal adenocarcinoma, and Liu et al. [9] discovered a substantial correlation between lung cancer and plasma metabolites. A series of metabolic events had also been occurring in RC, such as glycolysis, the tricarboxylic acid (TCA) cycle, glutamine metabolism, ATP production, and so on [10, 11]. In the TCA cycle, citric acid was utilized to synthesize fatty acids through the production of intracytoplasmic acetyl coenzyme A by ATP citrate lyase, facilitating the creation of lipid membranes in RC [12]. In addition, elevated levels of glycolytic metabolites and reduced levels of metabolites of oxidative phosphorylation were also revealed in RC [11]. As a result, it was indispensable for us to study the metabolic mechanisms involved in RC.

Mendelian randomization (MR) analysis was a method for determining the causal relationships between exposures and outcomes by using genetic variations [13]. Currently, MR analysis has been widely used to combine with expression quantitative trait loci (eQTLs) or cis‐eQTLs for identifying novel drug targets in various diseases [14, 15]. However, this combination had been rarely applied in RC. Hence in this article, we first integrated MR analysis with eQTLs and cis‐eQTLs to reveal novel therapeutic targets for RC and their related metabolic mechanisms.

## 2. Materials and Methods

### 2.1. Study Design

The study design of this article was mainly conducted following these steps: Step 0 (discovering and validating): SLC2A9 was first identified as a potential causal gene in the discovery dataset (IEU OpenGWAS eQTLs with the ID of eqtl‐a‐ENSG00000109667), and its role was subsequently validated across four independent datasets (eQTLGen Whole Blood cis‐eQTLs, GTEx Whole Blood cis‐eQTLs, TCGA‐KIRC dataset, and CPTAC‐KIRC dataset); Step 1: SLC2A9 eQTL as exposure and RC as outcome for two‐sample MR analysis; Step 2: 1400 metabolites as exposures and RC as outcome for two‐sample MR analyses; Step 3: SLC2A9 eQTL as exposure and 51 metabolites as outcomes for two‐sample MR analyses. Therein, Steps 1–3 were conducted to seek the related metabolic mechanisms of SLC2A9 eQTL involved in RC. The fundamental assumptions of MR [16] and the checklist for strengthening the reporting of observational studies in epidemiology (STROBE) [17] were strictly adhered to in this study.

### 2.2. Genome‐Wide Association Studies (GWASs) Data Sources

The IEU OpenGWAS dataset (https://gwas.mrcieu.ac.uk/) provided the genetic data of RC (ukb‐b‐1316, *N* = 463,010) and the 19,942 gene eQTL data (Accession ID format: eqtl‐a‐[Ensembl Gene ID]) from European individuals [18], including the SLC2A9 gene eQTL data (eqtl‐a‐ENSG00000109667, *N* = 26,609 sample sizes). Externally validated cis‐eQTLs were obtained from the eQTLGEN Whole Blood dataset (https://www.eqtlgen.org/cis-eqtls.html) [19] and the GTEx Whole Blood dataset (https://yanglab.westlake.edu.cn/software/smr/#DataResource) [20]. The GWAS Catalog dataset (https://www.ebi.ac.uk/gwas/home) provided the genetic data of 1400 metabolites with IDs of GCST90199621–GCST90201020 [21].

### 2.3. Instrumental Variables (IVs) Selection for Two‐Sample MR Analyses

For two‐sample MR analyses, when eQTLs were set as exposures, IVs were selected according to three conditions: ① *p*‐value threshold of 5 × 10^−8^; ② Linkage disequilibrium (LD) threshold of clumped kb = 10,000 kb and r^2^ = 0.001; ③ *F* statistic ≥10 for avoiding weak IV biases. When metabolites were set as exposures, IVs were selected according to three conditions: ① *p*‐value threshold of 1 × 10^−5^; ② LD threshold of clumped kb = 10,000 kb and r^2^ = 0.001; ③ *F* statistic ≥10 for avoiding weak IV biases.

### 2.4. Two‐Sample MR and Sensitivity Analyses

With the assistance of the R “TwoSampleMR” package, two‐sample MR analyses were performed by five methods, including the inverse square weighting (IVW), MR‐Egger, weighted model, weighted median, and simple model [22]. Therein, the IVW method was the main outcome of this study, and its *p*‐value below 0.05 was considered to be statistically significant [23]. Sensitivity analyses for two‐sample MR analyses mainly consisted of pleiotropy, heterogeneity, as well as leave‐one‐out analysis. Results might be impacted by horizontal pleiotropic effects for any other characteristic when its *p*‐value was below 0.05. Heterogeneity was tested by the Cochran *Q* statistic, with the *p*‐value threshold for significance set at 0.05 [24]. In addition, leave‐one‐out analysis was used to identify probable heterogeneous single nucleotide polymorphisms (SNPs).

### 2.5. Summary‐Data‐Based MR (SMR) and Sensitivity Analyses

For externally validated cis‐eQTLs from the eQTLGEN Whole Blood and the GTEx Whole Blood datasets, SMR analysis was conducted by the SMR 1.3.1 software, and its *p*‐value below 0.05 was considered to be statistically significant [14]. Sensitivity analysis for SMR analysis was conducted by the heterogeneity in dependent instruments (HEIDI) test in order to separate pleiotropy from linkage [15]. Moreover, scatter plots were applied to visualize the positive or negative correlations among exposures and outcomes.

### 2.6. External Validations in the TCGA and UALCAN Datasets

The TCGA‐KIRC dataset of 611 sample sizes (https://portal.gdc.cancer.gov/) [25] and the CPTAC‐KIRC dataset of 194 sample sizes (https://ualcan.path.uab.edu/analysis-prot.html) [26] were utilized to validate the mRNA and protein expression levels of SLC2A9 in RC, respectively. The TCGA‐KIRC patients with integrated clinical information were enrolled, including overall survival (OS), disease‐free survival (DFS), M stage, gender, T stage, race, age, grade, N stage, and stage. Differential analysis was performed by the R “limma” package with a *p*‐value threshold of 0.05. OS and DFS were conducted by the R “survival” package based on the median expression of SLC2A9 as cutoff values. Univariate and multivariate Cox regression analyses were performed to identify independent factors (M stage, gender, T stage, race, age, grade, N stage, stage, and SLC2A9) associated with OS prognosis in RC patients with a *p*‐value threshold of 0.05.

### 2.7. Statistical Analysis

All two‐sample MR analyses were conducted with the help of the R “TwoSampleMR” package in the R 4.3.2 software (http://www.r-project.org). SMR analysis was conducted by the SMR 1.3.1 software (https://yanglab.westlake.edu.cn/software/smr/). The threshold of a *p*‐value below 0.05 was considered statistically significant.

## 3. Results

### 3.1. Causal Effects of SLC2A9 eQTL on RC in the Discovering Dataset

**Figure 1:**
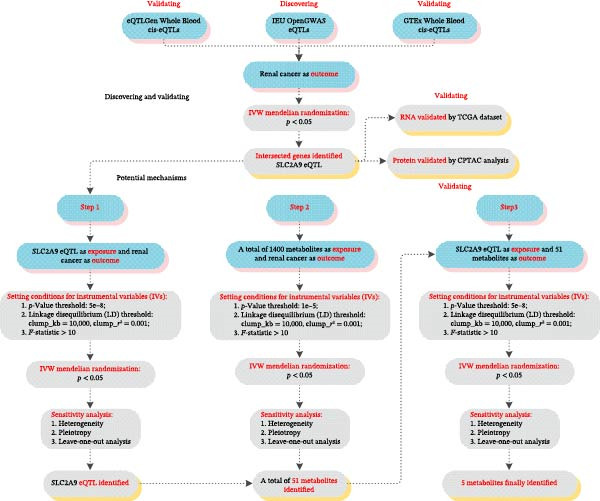
Illustration of the study design and workflow.

**Figure 2:**
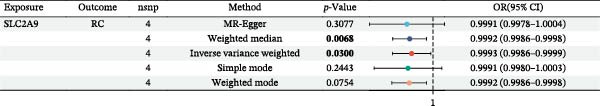
Forest plot to visualize the causal effects of SLC2A9 eQTL on RC in the discovering dataset (IEU OpenGWAS eQTLs).

Based on our study design in Figure 1, SLC2A9 was identified as an intersected gene from one discovering dataset (IEU OpenGWAS eQTLs) and four validating datasets (eQTLGen Whole Blood cis‐eQTLs, GTEx Whole Blood cis‐eQTLs, TCGA‐KIRC dataset, and CPTAC‐KIRC dataset). In the discovering dataset (IEU OpenGWAS eQTLs), two‐sample MR results showed that SLC2A9 eQTL was markedly associated with a low risk of RC (odds ratio [OR] = 0.9993, 95% confidence interval [CI] = 0.9986–0.9999, *p* = 0.030) within the IVW method (Figure 2). The *p*‐values for the heterogeneity and the pleiotropy tests were both above 0.05, indicating the robustness of the results. Its related forest map, scatter plot, and leave‐one‐out analysis were presented in Supporting Information 1: Figure S1.

### 3.2. External Validations in the eQTLGEN and GTEx Whole Blood Datasets

The eQTLGEN and GTEx Whole Blood datasets were utilized by us to externally validate the causal effects of SLC2A9 cis‐eQTL on RC by SMR analysis. Consistent with the results in the discovering dataset, SLC2A9 cis‐eQTL was also significantly linked with a low risk of RC in both the eQTLGEN dataset (OR = 0.9989, 95% CI = 0.9981–0.9997, *p* = 0.008) and the GTEx Whole Blood dataset (OR = 0.9989, 95% CI = 0.9980–0.9998, *p* = 0.016) (Figure 3A). The *p*‐values for the HEIDI tests were both above 0.05, indicating the robustness of the results. In addition, scatter plots showed the negative correlations among SLC2A9 cis‐eQTL and RC in both the eQTLGEN and GTEx Whole Blood datasets (Figure 3B,C).

Figure 3External validations of the causal effects of SLC2A9 cis‐eQTL on RC in the eQTLGEN and GTEx Whole Blood datasets by (A) forest map, (B) scatter plot in the eQTLGEN Whole Blood dataset, and (C) scatter plot in the GTEx Whole Blood dataset.

**Figure:**
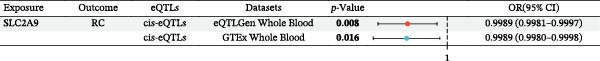
(A)

**Figure:**
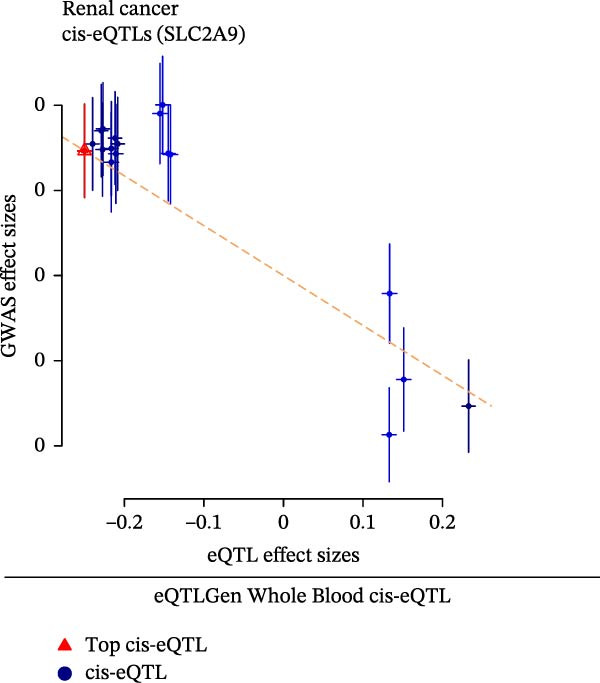
(B)

**Figure:**
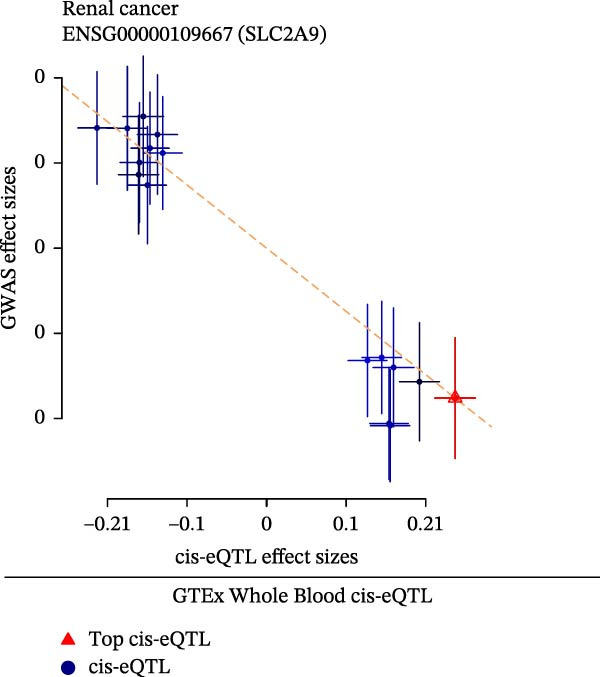
(C)

### 3.3. External Validations in the TCGA and UALCAN Datasets

The TCGA‐KIRC and the CPTAC‐KIRC datasets were utilized to validate the mRNA and protein expression levels of SLC2A9 in RC, respectively. Figure 4A showed that the mRNA levels of SLC2A9 were lower in RC tissues than in normal controls (*p* < 0.001) in the TCGA‐KIRC dataset. Meanwhile, high‐SLC2A9 groups showed better prognoses than low‐SLC2A9 groups for both OS (*p* < 0.001; Figure 4B) and DFS (*p* < 0.001; Figure 4C) in the TCGA‐KIRC dataset. Univariate and multivariate Cox regression analyses indicated that SLC2A9 could serve as an independent factor of OS in the TCGA‐KIRC dataset (both *p*  < 0.001; Figure 4D,E). Consistent with the mRNA expression levels, the protein expression levels of SLC2A9 were also lower in RC tissues (Figure 4F), different grade tissues (Figure 4G), and different stage tissues (Figure 4H) than in normal controls (*p* < 0.001) in the CPTAC‐KIRC dataset.

Figure 4External validations of the mRNA and protein expression levels of SLC2A9 in RC in the TCGA and UALCAN datasets; (A) boxplot of the mRNA expression of SLC2A9 in normal and tumor tissues in the TCGA‐KIRC dataset; (B) OS curve in the TCGA‐KIRC dataset; (C) DFS curve in the TCGA‐KIRC dataset; (D) univariate and (E) multivariate Cox regression analyses of SLC2A9 and clinicopathologic variables of OS in the TCGA‐KIRC dataset; (F) boxplot of the protein expression of SLC2A9 in normal and tumor tissues in the CPTAC‐KIRC dataset; (G) boxplot of the protein expression of SLC2A9 in normal and different grade tissues in the CPTAC‐KIRC dataset; and (H) boxplot of the protein expression of SLC2A9 in normal and different stage tissues in the CPTAC‐KIRC dataset.

**Figure:**
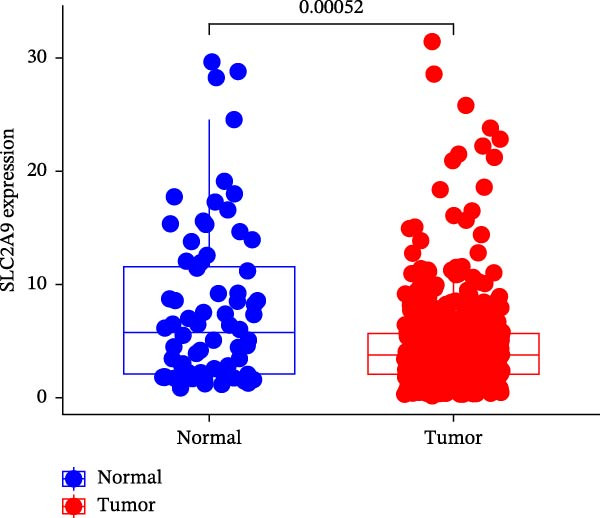
(A)

**Figure:**
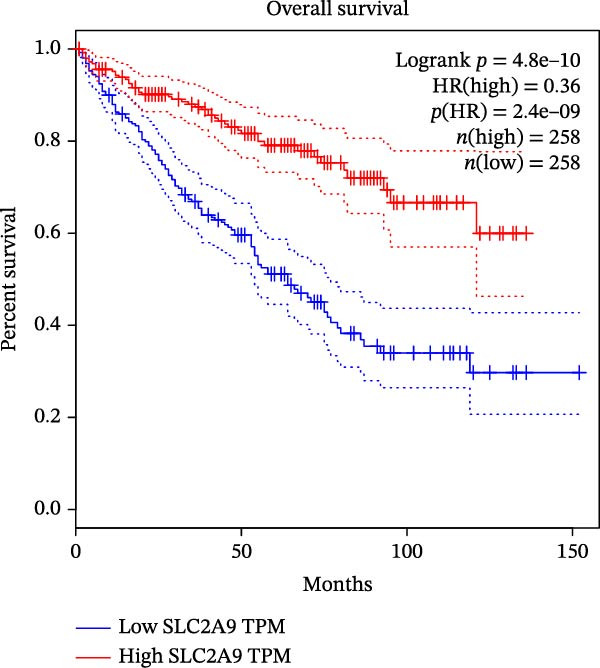
(B)

**Figure:**
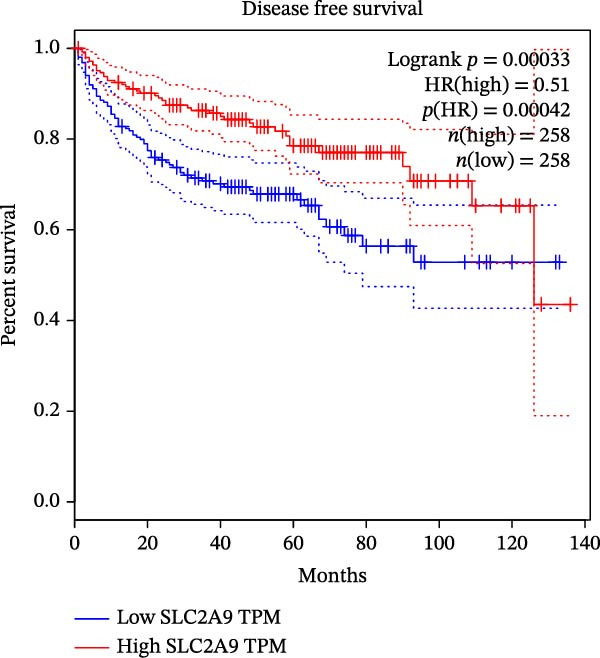
(C)

**Figure:**
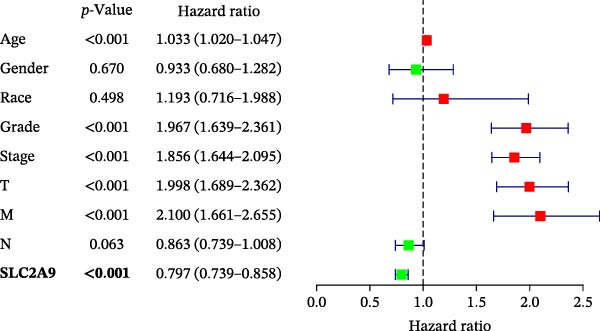
(D)

**Figure:**
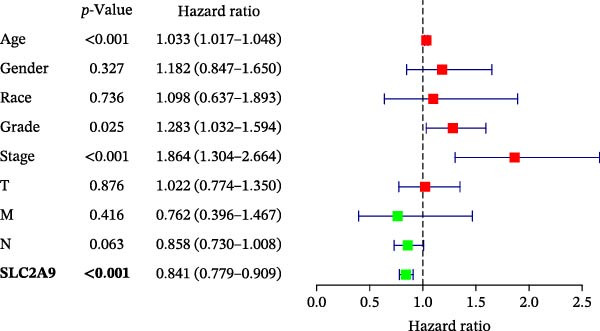
(E)

**Figure:**
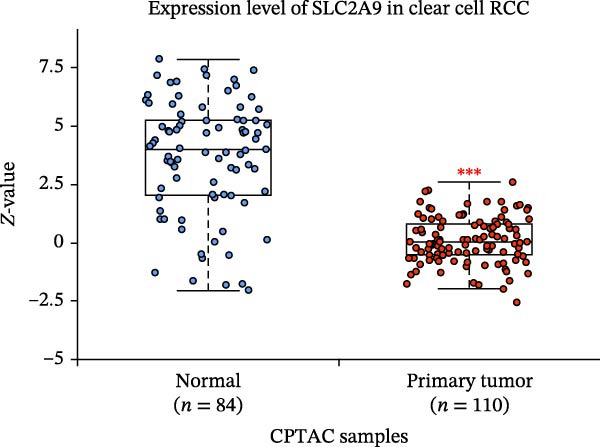
(F)

**Figure:**
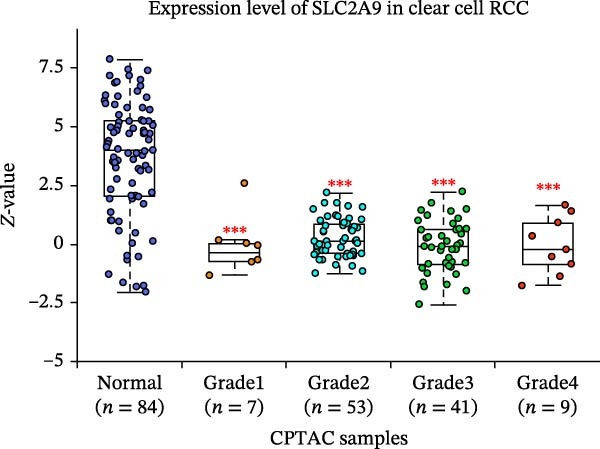
(G)

**Figure:**
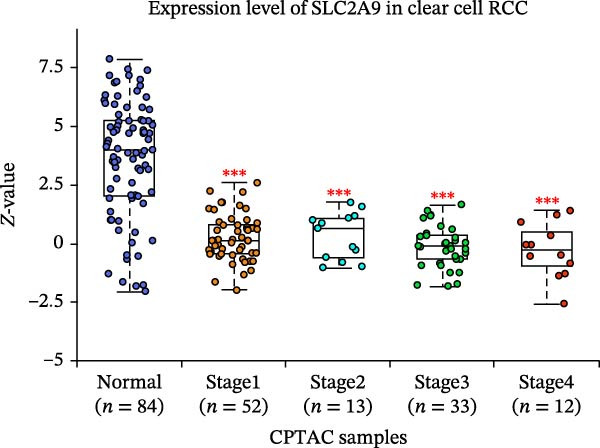
(H)

### 3.4. Causal Effects of SLC2A9 eQTL on Five Metabolites

Based on our study design in Figure 1, we finally identified a total of five metabolites for revealing the related metabolic mechanisms of SLC2A9 eQTL involved in RC, including GCST90199835 (5alpha‐androstan‐3beta, 17beta‐diol disulfate levels), GCST90200070 (1‐(1‐enyl‐palmitoyl)‐2‐palmitoleoyl‐GPC (P‐16:0/16:1) levels), GCST90200083 (1‐(1‐enyl‐palmitoyl)‐2‐palmitoyl‐GPC (P‐16:0/16:0) levels), GCST90200270 (2‐methoxyhydroquinone sulfate (1) levels), and GCST90200537 (X‐16087 levels).

**Figure 5:**
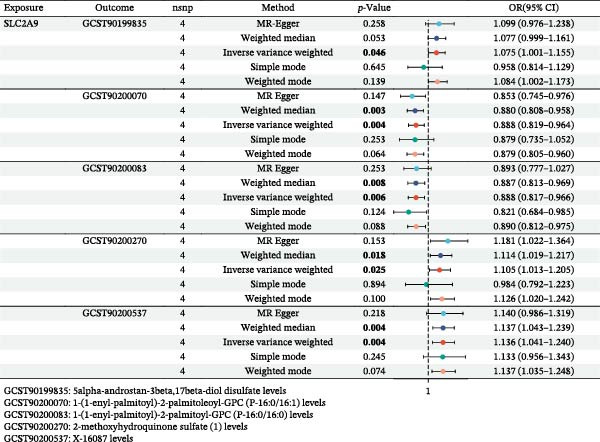
Forest plot to visualize the causal effects of SLC2A9 eQTL on five metabolites.

Figure 5 showed that SLC2A9 eQTL was significantly linked with increased levels of three metabolites within the IVW method by two‐sample MR results, containing 5alpha‐androstan‐3beta, 17beta‐diol disulfate levels (GCST90199835, OR = 1.075, 95% CI = 1.001–1.155, *p* = 0.048), 2‐methoxyhydroquinone sulfate (1) levels (GCST90200270, OR = 1.105, 95% CI = 1.013–1.205, *p* = 0.025), and X‐16087 levels (GCST90200537, OR = 1.136, 95% CI = 1.041–1.240, *p* = 0.004). SLC2A9 eQTL was also markedly associated with decreased levels of two metabolites within the IVW method by two‐sample MR results, including 1‐(1‐enyl‐palmitoyl)‐2‐palmitoleoyl‐GPC (P‐16:0/16:1) levels (GCST90200070, OR = 0.888, 95% CI = 0.819–0.964, *p* = 0.004) and 1‐(1‐enyl‐palmitoyl)‐2‐palmitoyl‐GPC (P‐16:0/16:0) levels (GCST90200083, OR = 0.888, 95% CI = 0.817–0.966, *p* = 0.006). Their *p*‐values for the heterogeneity and the pleiotropy tests were both above 0.05, indicating the stability of our results. Their related forest map, scatter plot, and leave‐one‐out analysis were presented in Supporting Information 2: Figure S2. Supporting Information 3: Table S1 displayed the detailed data of the causal effects of SLC2A9 eQTL on five metabolites by *p*‐values and false discovery rate (FDR).

### 3.5. Causal Effects of Five Metabolites on RC

**Figure 6:**
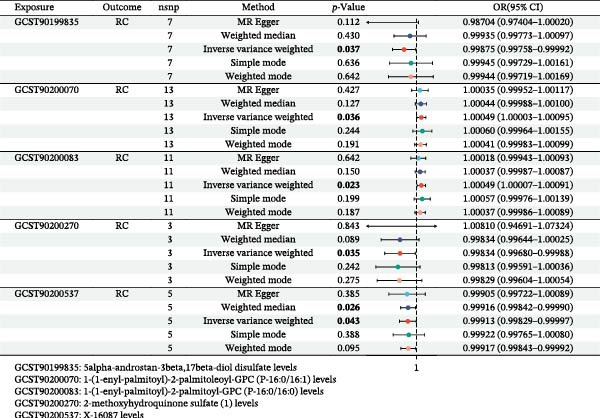
Forest plot to visualize the causal effects of five metabolites on RC.

**Figure 7:**
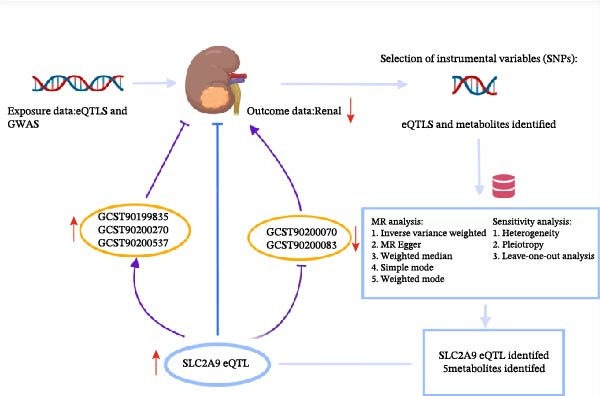
Schematic diagram of the role of SLC2A9 in RC and its related metabolic mechanisms.

Figure 6 showed that three metabolites were markedly associated with decreased risks of RC within the IVW method by two‐sample MR results, including 5alpha‐androstan‐3beta, 17beta‐diol disulfate levels (GCST90199835, OR = 0.99875, 95% CI = 0.99758–0.99992, *p* = 0.037), 2‐methoxyhydroquinone sulfate (1) levels (GCST90200270, OR = 0.99834, 95% CI = 0.99680–0.99988, *p* = 0.035), and X‐16087 levels (GCST90200537, OR = 0.99913, 95% CI = 0.99829–0.99997, *p* = 0.043). Two metabolites were also significantly linked with increased risks of RC within the IVW method by two‐sample MR results, containing 1‐(1‐enyl‐palmitoyl)‐2‐palmitoleoyl‐GPC (P‐16:0/16:1) levels (GCST90200070, OR = 1.00049, 95% CI = 1.00003–1.00095, *p* = 0.036), and 1‐(1‐enyl‐palmitoyl)‐2‐palmitoyl‐GPC(P‐16:0/16:0) levels (GCST90200083, OR = 0.1.00049, 95% CI = 1.00007–1.00091, *p* = 0.023). Their *p*‐values for the heterogeneity and the pleiotropy tests were both above 0.05, indicating the stability of our results. Their related forest map, scatter plot, and leave‐one‐out analysis were presented in Supporting Information 4: Figure S3. Supporting Information 5: Table S2 presented the detailed data of the causal effects of five metabolites on RC by *p*‐values and FDR. Taken together, the antitumor roles of SLC2A9 in RC and its preliminarily identified metabolic mechanisms for these five metabolites were summarized in Figure 7.

## 4. Discussion

MR analysis integrated with eQTLs and cis‐eQTLs was first utilized in this article by us to reveal the causal effects among SLC2A9 and RC, along with shedding light on their related metabolic mechanisms. Our results found that SLC2A9 eQTL was markedly associated with low risks of RC in the IEU OpenGWAS eQTLs dataset (discovering), validated by SLC2A9 cis‐eQTL in the eQTLGEN and GTEx Whole Blood datasets. The TCGA‐KIRC and the CPTAC‐KIRC datasets were utilized to validate the mRNA and protein expression levels of SLC2A9 in RC, confirming its antitumor roles in RC. To further reveal the related metabolic mechanisms of SLC2A9 eQTL involved in RC, 1400 metabolites were enrolled as mediators, and a total of five metabolites were finally identified, including GCST90199835 (5alpha‐androstan‐3beta, 17beta‐diol disulfate levels), GCST90200070 (1‐(1‐enyl‐palmitoyl)‐2‐palmitoleoyl‐GPC (P‐16:0/16:1) levels), GCST90200083 (1‐(1‐enyl‐palmitoyl)‐2‐palmitoyl‐GPC (P‐16:0/16:0) levels), GCST90200270 (2‐methoxyhydroquinone sulfate (1) levels), and GCST90200537 (X‐16087 levels). As no statistically robust metabolic mediators were identified after FDR correction, the five metabolites were presented only as preliminary candidates requiring further investigation.

SLC2A9 belonged to a member of Class II of the promoting glucose transporter family and was reported to be a transporter protein found in the kidney, liver, chondrocytes, and so on [27, 28]. It played a significant role in the proximal renal tubule reabsorption and secretion, as well as the maintenance of a steady serum uric acid concentration, which was a key factor in plasma uric acid levels and the development of gout [29–31]. In recent years, SLC2A9 and its regulation of uric acid levels had been strongly linked with tumors. Some studies had found significant associations among SLC2A9 and lung adenocarcinomas, prostate cancer, and so on [32, 33]. Moreover, high plasma urate was also found to be linked with increased cancer risks and elevated all‐cause mortality [34]. However, the roles of SLC2A9 in RC had not yet been investigated in any research. Our results first showed that SLC2A9 eQTL was markedly associated with low risks of RC, exerting an antitumor role.

Several potential reasons could explain the significant role of SLC2A9 in RC. First, the urate regulated by SLC2A9 [35, 36] reduced oxidative stress‐induced apoptosis by eliminating reactive oxygen species (ROS) linked to tumor growth, which in turn allowed cancer cells to proliferate and survive [37, 38]. Second, low expression of SLC2A9 could result in an overload of uric acid entering the cell, which would activate NF‐KB and the proinflammatory mediators MCP‐1 and COX‐2, causing low‐grade inflammation [39]. This microenvironment with sustained low‐grade inflammation was conducive to the transformation of normal cells into cancer cells [38]. Moreover, sustained activation of NF‐KB would promote the activation of the renal IMD‐NF‐KB pathway, which further led to more uric acid entering the cells to exacerbate the process [40]. As we know, inflammation was significantly linked with RC via fostering tumor prognosis, progression, and immune evasion [41]. Consequently, inflammation‐related markers or signatures had been regarded as independent predictors in RC patients [42]. Third, as a regulator of SLC2A9 expression in vivo, HNF4α (nuclear factor 4α) antagonized the function of the hypoxia‐inducible factor HIF1/2α in metabolic reprogramming to inhibit tumor growth [43]. All of the abovementioned factors partly shed light on the antitumor roles of SLC2A9 in RC.

To further reveal the related metabolic mechanisms of SLC2A9 eQTL involved in RC, a total of five metabolites were finally identified. Therein, the 1‐(1‐enyl‐palmitoyl)‐2‐palmitoleoyl‐GPC (P‐16:0/16:1) levels and 1‐(1‐enyl‐palmitoyl)‐2‐palmitoyl‐GPC (P‐16:0/16:0) levels could increase RC risks, while the 5alpha‐androstan‐3beta, 17beta‐diol disulfate levels, 2‐methoxyhydroquinone sulfate (1) levels, and X‐16087 levels could reduce RC risks. As reported, the 1‐(1‐enyl‐palmitoyl)‐2‐palmitoleoyl‐GPC (P‐16:0/16:1) levels and 1‐(1‐enyl‐palmitoyl)‐2‐palmitoyl‐GPC (P‐16:0/16:0) levels were metabolites of protein palmitoylation, which was the most prevalent lipoylation modification in action [44]. Hundreds of mammalian palmitoylated proteins had been identified, and most of them were reported to be associated with tumor survival and progression [45, 46]. In our study, these two palmitoylated metabolites were also found to be closely related to RC, which deserved further deep exploration. We further hypothesized that impaired SLC2A9 function disrupted intracellular urate homeostasis, potentially altering redox balance in proximal tubules [47]. This oxidative stress might downregulate DHHC9 palmitoyltransferase activity, reducing protein palmitoylation and diverting palmitoyl‐CoA toward glycerophospholipid synthesis [48]. The accumulated palmitate derivatives (P‐16:0/16:1 and P‐16:0/16:0 GPCs) could then incorporate into RC membranes, modifying lipid raft composition and SLC2A9 trafficking [49, 50], thus linking SLC2A9‐mediated urate transport with RC survival.

MR mediation analysis had the capacity to enhance comprehension of causation and pinpoint intermediary factors as possible intervention targets [51]. For more accurate causal analysis, several studies had recently expanded the application of this analysis method [52]. Xu et al. [53] explored SGLT2 and choline metabolism associated with heart disease by using the MR mediation analysis. Liu et al. [54] investigated the causal relationship between nonalcoholic fatty liver disease and polycystic ovary syndrome through mediation analysis. In this article, MR mediation analysis showed that SLC2A9 indirectly inhibited RC by promoting 5alpha‐androstan‐3beta, 17beta‐diol disulfate levels, 2‐methoxyhydroquinone sulfate (1) levels, and X‐16087 levels, while indirectly inhibiting RC by suppressing 1‐(1‐enyl‐palmitoyl)‐2‐palmitoleoyl‐GPC (P‐16:0/16:1) levels and 1‐(1‐enyl‐palmitoyl)‐2‐palmitoyl‐GPC (P‐16:0/16:0) levels. To summarize, our results showed that these five metabolites could bridge the causal effects between SLC2A9 eQTL and RC.

We first integrated MR analysis with eQTLs and cis‐eQTLs to reveal the causal effects among SLC2A9 and RC, along with shedding light on their related metabolic mechanisms. SLC2A9 was identified as an intersected gene from one discovering dataset (IEU OpenGWAS eQTLs) and four validating datasets (eQTLGen Whole Blood cis‐eQTLs, GTEx Whole Blood cis‐eQTLs, TCGA‐KIRC dataset, and CPTAC‐KIRC dataset). The eQTLGEN and GTEx Whole Blood datasets validated the causal effects among SLC2A9 cis‐eQTL and RC, reducing RC risks. The TCGA‐KIRC and the CPTAC‐KIRC datasets validated the mRNA and protein expression levels of SLC2A9 in RC, confirming its antitumor roles in RC. Finally, a total of five metabolites were identified to reveal the related metabolic mechanisms of SLC2A9 eQTL involved in RC.

There were a few limitations to our study. First, the participants were all European, and the results of the study might not be relevant to other areas or ethnic groups with certain differences in prevalence. Second, the function of genes and proteins not only depended on the level of gene expression but also changed in subcellular localization and posttranslational modification. Moreover, the overall transcription and translation state of cells could also affect gene expression. Third, more existing MR (e.g., MR‐RAPS) and cis‐MR methods (e.g., mr‐mvpcgmm, cis‐MRcML, and cis‐MRBEE) should be conducted for confirming the robustness of our results. Last but not least, as no statistically robust metabolic mediators were identified after FDR correction, the five metabolites were presented only as preliminary candidates requiring further investigation for their roles via in vivo and in vitro experiments.

## 5. Conclusions

MR analysis integrated with eQTLs and cis‐eQTLs was first utilized in this article by us to reveal the causal effects among SLC2A9 and RC, along with shedding light on their related metabolic mechanisms. Our results indicated that SLC2A9 eQTL or cis‐eQTL was markedly associated with low risks of RC, playing an antitumor role. Moreover, we preliminarily identified a total of five metabolites for revealing the related metabolic mechanisms of SLC2A9 eQTL involved in RC, requiring further investigation. This study provided a new idea to explore the intersection of RC and metabolites, inspiring future therapeutic target development in RC.

**Nomenclature**
MR: Mendelian randomization
eQTLs: Expression quantitative trait loci
RC: Renal cancer
GWAS: Genome‐wide association study
SMR: Summary‐data‐based Mendelian randomization study
ccRCC: Clear cell renal cell carcinoma
pRCC: Papillary RCC
chRCC: Chromophobe RCC
CDRCC: Collecting duct RCC
srRCC: Sarcomatoid RCC
TCA: Tricarboxylic acid
OS: Overall survival
DFS: Disease‐free survival.

## Author Contributions

**Yi Wang and Yu Song**: manuscript writing/editing/revision. **Yi Wang and Hao Ji**: data analysis. **Yi Wang, Yu Song, and Hao Ji**: data collection or management. **Bingye Zhu and Huyang Xie**: protocol/project development.

## Funding

The authors have nothing to report.

## Disclosure

All the co‐authors agreed to publish the final version of this manuscript.

## Ethics Statement

The authors have nothing to report.

## Consent

The authors have nothing to report.

## Conflicts of Interest

The authors declare no conflicts of interest.

## Supporting Information

Additional supporting information can be found online in the Supporting Information section.

## Supporting information


**Supporting Information 1** Figure S1: The (A) forest map, (B) scatter plot, and (C) leave‐one‐out analysis to visualize the causal effects of SLC2A9 eQTL on RC in the discovering dataset (IEU OpenGWAS eQTLs).

**Supporting Information 2** Figure S2: The forest map, scatter plot, and leave‐one‐out analysis to visualize the causal effects of SLC2A9 eQTL on five metabolites, including (A–C) GCST90199835 (5alpha‐androstan‐3beta,17beta‐diol disulfate levels); (D–F) GCST90200070 (1‐(1‐enyl‐palmitoyl)‐2‐palmitoleoyl‐GPC (P‐16:0/16:1) levels); (G–I) GCST90200083 (1‐(1‐enyl‐palmitoyl)‐2‐palmitoyl‐GPC (P‐16:0/16:0) levels); (J–L) GCST90200270 (2‐methoxyhydroquinone sulfate (1) levels); and (M–O) GCST90200537 (X‐16087 levels).

**Supporting Information 3** Table S1: The causal effects of SLC2A9 eQTL on five metabolites by *p*‐values and false discovery rate (FDR).

**Supporting Information 4** Figure S3. The forest map, scatter plot, and leave‐one‐out analysis to visualize the causal effects of five metabolites on RC, including (A–C) GCST90199835 (5alpha‐androstan‐3beta,17beta‐diol disulfate levels); (D–F) GCST90200070 (1‐(1‐enyl‐palmitoyl)‐2‐palmitoleoyl‐GPC (P‐16:0/16:1) levels); (G–I) GCST90200083 (1‐(1‐enyl‐palmitoyl)‐2‐palmitoyl‐GPC (P‐16:0/16:0) levels); (J–L) GCST90200270 (2‐methoxyhydroquinone sulfate (1) levels); and (M–O) GCST90200537 (X‐16087 levels).

**Supporting Information 5** Table S2: The causal effects of five metabolites on RC by *p*‐values and false discovery rate (FDR).

## Data Availability

The IEU OpenGWAS dataset (https://gwas.mrcieu.ac.uk/) provided the genetic data of RC (ukb‐b‐1316) and the 19,942 gene eQTL data (Accession ID format: eqtl‐a‐[Ensembl Gene ID]). Externally validated cis‐eQTLs were obtained from the eQTLGEN Whole Blood dataset (https://www.eqtlgen.org/cis-eqtls.html) and the GTEx Whole Blood dataset (https://yanglab.westlake.edu.cn/software/smr/#DataResource). The GWAS Catalog dataset (https://www.ebi.ac.uk/gwas/home) provided the genetic data of 1400 metabolites with IDs of GCST90199621–GCST90201020. The TCGA‐KIRC dataset (https://portal.gdc.cancer.gov/) and the CPTAC‐KIRC dataset (https://ualcan.path.uab.edu/analysis-prot.html) were utilized to validate the RNA and protein levels of SLC2A9 in RC, respectively.
