# Supplementary figures and images for: Mendelian Randomization Identified SLC2A9 as a Novel cis‐eQTL‐Mediated Susceptibility Gene in Suppressing Renal Cancer and Its Related Metabolic Mechanisms
Source: Mediators Inflamm. 2026 Mar 16;2026:5817314. doi: 10.1155/mi/5817314 (PMC13140309; doi:10.1155/mi/5817314)

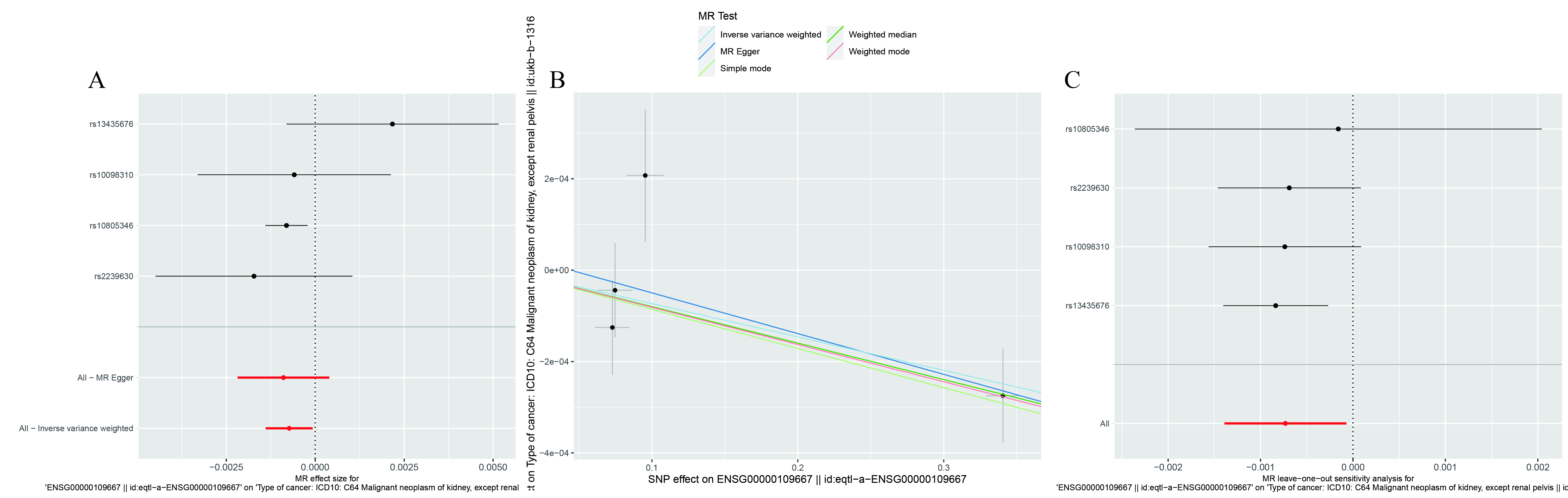

Supplement: Supplementary file 1 — Supporting Information 1 Figure S1: The (A) forest map, (B) scatter plot, and (C) leave‐one‐out analysis to visualize the causal effects of SLC2A9 eQTL on RC in the discovering dataset (IEU OpenGWAS eQTLs). [file MI-2026-5817314-s004.tif]
