# Supplementary material for: Mendelian Randomization Identified SLC2A9 as a Novel cis‐eQTL‐Mediated Susceptibility Gene in Suppressing Renal Cancer and Its Related Metabolic Mechanisms
Source: Mediators Inflamm. 2026 Mar 16;2026:5817314. doi: 10.1155/mi/5817314 (PMC13140309; doi:10.1155/mi/5817314)
Supplement: Supplementary file 3 — Supporting Information 3 Table S1: The causal effects of SLC2A9 eQTL on five metabolites by p‐values and false discovery rate (FDR). [file MI-2026-5817314-s003.docx]

**Table S1**. The causal effects of SLC2A9 eQTL on five metabolites by p-values and false discovery rate (FDR).

| **Exposure** | **Outcome** | **Method** | **Nsnp** | **Beta** | **SE** | **Pval** | **lo_ci** | **up_ci** | **OR** | **OR_lci95** | **OR_uci95** | **FDR** |
| --- | --- | --- | --- | --- | --- | --- | --- | --- | --- | --- | --- | --- |
| SLC2A9 | GCST90199835 | MR Egger | 4 | 0.094752 | 0.060559 | 0.258133 | -0.023943 | 0.213448 | 1.099387 | 0.976341 | 1.237939 | 0.322666 |
|  |  | Weighted median | 4 | 0.074246 | 0.038315 | 0.052652 | -0.000852 | 0.149344 | 1.077072 | 0.999148 | 1.161072 | 0.131631 |
|  |  | Inverse variance weighted | 4 | 0.07268 | 0.036471 | 0.046282 | 0.001197 | 0.144163 | 1.075386 | 1.001197 | 1.155072 | 0.131631 |
|  |  | Simple mode | 4 | -0.042587 | 0.083444 | 0.644973 | -0.206137 | 0.120962 | 0.958307 | 0.813722 | 1.128583 | 0.644973 |
|  |  | Weighted mode | 4 | 0.080699 | 0.040359 | 0.139386 | 0.001596 | 0.159802 | 1.084045 | 1.001598 | 1.173279 | 0.23231 |
| SLC2A9 | GCST90200070 | MR Egger | 4 | -0.15935 | 0.06893 | 0.14695 | -0.29446 | -0.02425 | 0.85269 | 0.74494 | 0.97604 | 0.18369 |
|  |  | Weighted median | 4 | -0.12822 | 0.04364 | 0.00330 | -0.21374 | -0.04269 | 0.87966 | 0.80756 | 0.95821 | 0.01087 |
|  |  | Inverse variance weighted | 4 | -0.11841 | 0.04152 | 0.00435 | -0.19979 | -0.03702 | 0.88833 | 0.81890 | 0.96365 | 0.01087 |
|  |  | Simple mode | 4 | -0.12872 | 0.09131 | 0.25344 | -0.30769 | 0.05026 | 0.87922 | 0.73514 | 1.05154 | 0.25344 |
|  |  | Weighted mode | 4 | -0.12872 | 0.04471 | 0.06359 | -0.21635 | -0.04108 | 0.87922 | 0.80545 | 0.95975 | 0.10598 |
| SLC2A9 | GCST90200083 | MR Egger | 4 | -0.11279 | 0.07100 | 0.25309 | -0.25194 | 0.02637 | 0.89334 | 0.77729 | 1.02672 | 0.25309 |
|  |  | Weighted median | 4 | -0.11941 | 0.04468 | 0.00753 | -0.20698 | -0.03183 | 0.88745 | 0.81303 | 0.96867 | 0.01883 |
|  |  | Inverse variance weighted | 4 | -0.11843 | 0.04276 | 0.00561 | -0.20224 | -0.03463 | 0.88831 | 0.81690 | 0.96597 | 0.01883 |
|  |  | Simple mode | 4 | -0.19727 | 0.09311 | 0.12433 | -0.37977 | -0.01478 | 0.82097 | 0.68402 | 0.98533 | 0.15541 |
|  |  | Weighted mode | 4 | -0.11666 | 0.04665 | 0.08766 | -0.20810 | -0.02522 | 0.88989 | 0.81213 | 0.97510 | 0.14611 |
| SLC2A9 | GCST90200270 | MR Egger | 4 | 0.16604 | 0.07366 | 0.15290 | 0.02167 | 0.31041 | 1.18062 | 1.02191 | 1.36398 | 0.19112 |
|  |  | Weighted median | 4 | 0.10753 | 0.04536 | 0.01776 | 0.01862 | 0.19643 | 1.11352 | 1.01880 | 1.21705 | 0.06263 |
|  |  | Inverse variance weighted | 4 | 0.09942 | 0.04437 | 0.02505 | 0.01245 | 0.18638 | 1.10453 | 1.01253 | 1.20488 | 0.06263 |
|  |  | Simple mode | 4 | -0.01611 | 0.11076 | 0.89360 | -0.23319 | 0.20098 | 0.98402 | 0.79200 | 1.22260 | 0.89360 |
|  |  | Weighted mode | 4 | 0.11832 | 0.05027 | 0.09998 | 0.01979 | 0.21686 | 1.12561 | 1.01998 | 1.24217 | 0.16663 |
| SLC2A9 | GCST90200537 | MR Egger | 4 | 0.13140 | 0.07417 | 0.21846 | -0.01397 | 0.27677 | 1.14043 | 0.98613 | 1.31887 | 0.24459 |
|  |  | Weighted median | 4 | 0.12821 | 0.04409 | 0.00364 | 0.04179 | 0.21464 | 1.13680 | 1.04268 | 1.23941 | 0.01062 |
|  |  | Inverse variance weighted | 4 | 0.12769 | 0.04466 | 0.00425 | 0.04016 | 0.21523 | 1.13620 | 1.04097 | 1.24014 | 0.01062 |
|  |  | Simple mode | 4 | 0.12518 | 0.08672 | 0.24459 | -0.04479 | 0.29516 | 1.13336 | 0.95619 | 1.34335 | 0.24459 |
|  |  | Weighted mode | 4 | 0.12825 | 0.04767 | 0.07438 | 0.03482 | 0.22168 | 1.13684 | 1.03543 | 1.24817 | 0.12397 |
