# Supplementary material for: Mendelian Randomization Identified SLC2A9 as a Novel cis‐eQTL‐Mediated Susceptibility Gene in Suppressing Renal Cancer and Its Related Metabolic Mechanisms
Source: Mediators Inflamm. 2026 Mar 16;2026:5817314. doi: 10.1155/mi/5817314 (PMC13140309; doi:10.1155/mi/5817314)
Supplement: Supplementary file 5 — Supporting Information 5 Table S2: The causal effects of five metabolites on RC by p‐values and false discovery rate (FDR). [file MI-2026-5817314-s005.docx]

**Table S2**. The causal effects of five metabolites on RC by p-values and false discovery rate (FDR).

| **Exposure** | **Outcome** | **Method** | **Nsnp** | **Beta** | **SE** | **Pval** | **lo_ci** | **up_ci** | **OR** | **OR_lci95** | **OR_uci95** | **FDR** |
| --- | --- | --- | --- | --- | --- | --- | --- | --- | --- | --- | --- | --- |
| GCST90199835 | RC | MR Egger | 7 | -0.01305 | 0.00676 | 0.11152 | -0.02630 | 0.00020 | 0.98704 | 0.97404 | 1.00020 | 0.27880 |
|  |  | Weighted median | 7 | -0.00065 | 0.00083 | 0.42981 | -0.00227 | 0.00097 | 0.99935 | 0.99773 | 1.00097 | 0.64206 |
|  |  | Inverse variance weighted | 7 | -0.00125 | 0.00060 | 0.03681 | -0.00242 | -0.00008 | 0.99875 | 0.99758 | 0.99992 | 0.18406 |
|  |  | Simple mode | 7 | -0.00055 | 0.00110 | 0.63606 | -0.00271 | 0.00161 | 0.99945 | 0.99729 | 1.00161 | 0.64206 |
|  |  | Weighted mode | 7 | -0.00056 | 0.00115 | 0.64206 | -0.00282 | 0.00169 | 0.99944 | 0.99719 | 1.00169 | 0.64206 |
| GCST90200070 | RC | MR Egger | 13 | 0.00035 | 0.00042 | 0.42711 | -0.00048 | 0.00117 | 1.00035 | 0.99952 | 1.00117 | 0.42711 |
|  |  | Weighted median | 13 | 0.00044 | 0.00029 | 0.12728 | -0.00012 | 0.00100 | 1.00044 | 0.99988 | 1.00100 | 0.30446 |
|  |  | Inverse variance weighted | 13 | 0.00049 | 0.00023 | 0.03603 | 0.00003 | 0.00094 | 1.00049 | 1.00003 | 1.00095 | 0.18013 |
|  |  | Simple mode | 13 | 0.00060 | 0.00049 | 0.24357 | -0.00036 | 0.00155 | 1.00060 | 0.99964 | 1.00155 | 0.30446 |
|  |  | Weighted mode | 13 | 0.00041 | 0.00030 | 0.19068 | -0.00017 | 0.00099 | 1.00041 | 0.99983 | 1.00099 | 0.30446 |
| GCST90200083 | RC | MR Egger | 11 | 0.00018 | 0.00038 | 0.64223 | -0.00057 | 0.00093 | 1.00018 | 0.99943 | 1.00093 | 0.64223 |
|  |  | Weighted median | 11 | 0.00037 | 0.00026 | 0.14995 | -0.00013 | 0.00087 | 1.00037 | 0.99987 | 1.00087 | 0.24867 |
|  |  | Inverse variance weighted | 11 | 0.00049 | 0.00022 | 0.02346 | 0.00007 | 0.00091 | 1.00049 | 1.00007 | 1.00091 | 0.11728 |
|  |  | Simple mode | 11 | 0.00057 | 0.00042 | 0.19894 | -0.00024 | 0.00139 | 1.00057 | 0.99976 | 1.00139 | 0.24867 |
|  |  | Weighted mode | 11 | 0.00037 | 0.00026 | 0.18735 | -0.00014 | 0.00089 | 1.00037 | 0.99986 | 1.00089 | 0.24867 |
| GCST90200270 | RC | MR Egger | 3 | 0.00806 | 0.03195 | 0.84258 | -0.05455 | 0.07068 | 1.00810 | 0.94691 | 1.07324 | 1.00000 |
|  |  | Weighted median | 3 | -0.00166 | 0.00097 | 0.08871 | -0.00357 | 0.00025 | 0.99834 | 0.99644 | 1.00025 | 2.00000 |
|  |  | Inverse variance weighted | 3 | -0.00166 | 0.00079 | 0.03486 | -0.00320 | -0.00012 | 0.99834 | 0.99680 | 0.99988 | 3.00000 |
|  |  | Simple mode | 3 | -0.00187 | 0.00114 | 0.24199 | -0.00410 | 0.00036 | 0.99813 | 0.99591 | 1.00036 | 4.00000 |
|  |  | Weighted mode | 3 | -0.00171 | 0.00115 | 0.27525 | -0.00397 | 0.00054 | 0.99829 | 0.99604 | 1.00054 | 5.00000 |
| GCST90200537 | RC | MR Egger | 5 | -0.00095 | 0.00094 | 0.38534 | -0.00278 | 0.00088 | 0.99905 | 0.99722 | 1.00089 | 0.38774 |
|  |  | Weighted median | 5 | -0.00084 | 0.00038 | 0.02568 | -0.00159 | -0.00010 | 0.99916 | 0.99842 | 0.99990 | 0.10799 |
|  |  | Inverse variance weighted | 5 | -0.00087 | 0.00043 | 0.04320 | -0.00171 | -0.00003 | 0.99913 | 0.99829 | 0.99997 | 0.10799 |
|  |  | Simple mode | 5 | -0.00078 | 0.00080 | 0.38774 | -0.00235 | 0.00080 | 0.99922 | 0.99765 | 1.00080 | 0.38774 |
|  |  | Weighted mode | 5 | -0.00083 | 0.00038 | 0.09492 | -0.00157 | -0.00008 | 0.99917 | 0.99843 | 0.99992 | 0.15821 |
